# RSV Disease Burden in Primary Care in Italy: A Multi‐Region Pediatric Study, Winter Season 2022–2023

**DOI:** 10.1111/irv.13282

**Published:** 2024-04-15

**Authors:** Michela Scarpaci, Sara Bracaloni, Enrica Esposito, Luigi De Angelis, Francesco Baglivo, Beatrice Casini, Donatella Panatto, Matilde Ogliastro, Daniela Loconsole, Maria Chironna, Elena Pariani, Laura Pellegrinelli, Elisabetta Pandolfi, Ileana Croci, Caterina Rizzo, RSVComNet Italia, Mauro Pistello, Tommaso Cosci, Francesca Centrone, Giancarlo Icardi, Piero Luigi Lai, Carola Minet, Giada Garzillo, Bianca Roncan, Sara Tardito, Marta Crocetti, Andrea Orsi, Elvira Massaro, Giulia Lindaros, Cristina Russo, Cristina Galli, Arlinda Seiti, Sheila Santisteban, Silvana Castaldi, Sandro Binda, Paola Guidotti, Patrizia Rogari, Arianna Passoni, Luigi Greco, Micaela Foco, Claudia Pontesilli, Michele Valente, Laura Reali, Laura Venuti, Valentina Grimaldi, Donatella Morano, Carlo Federico Perno, Innocenza Rafele, Fabrizio Piperno, Giovanni Capaldi, Daniela Damiani, Vincenzo Frappampina, Stefania Frau, Nunzio Guglielmi, Lucia Peccarisi, Monica Pepe, Giuseppe Ragnatela, Cristoforo Vania, Massimo Ciampolini, Marco Gucci, Vittorio Tarabella, Fiorella Di Dia, Allegra Boni, Donatella Gazzarrini, Santina Torri, Giulia Camici, Claudio Memmini, Donata Mora, Daniela Gaezza, Patrizia Benedetti, Marica Del Fiorentino, Fabiola Salvetti, Benedetta Marchi, Giuliano Maggiani, Elisabetta Maria Bellino, Stefano Castelli, Luigina Vaccarone, Silvia Cecchetti, Alberto Eugenio Tozzi

**Affiliations:** ^1^ Department of Translational Research and New Technologies in Medicine and Surgery University of Pisa Pisa Italy; ^2^ Department of Health Sciences University of Genoa Genoa Italy; ^3^ Hygiene Section, Department of Interdisciplinary Medicine University of Bari “A. Moro” Bari Italy; ^4^ Department of Biomedical Sciences for Health University of Milan Milan Italy; ^5^ Predictive and Preventive Medicine Research Unit Bambino Gesù Children's Hospital, IRCCS Rome Italy

**Keywords:** acute respiratory infections, primary care, respiratory infection surveillance, RSV

## Abstract

**Introduction:**

Human respiratory syncytial virus (RSV) is one of the most frequent causes of respiratory infections in children under 5 years of age, but its socioeconomic impact and burden in primary care settings is still little studied.

**Methods:**

During the 2022/2023 winter season, 55 pediatricians from five Italian regions participated in our community‐based study. They collected a nasal swab for RSV molecular test from 650 patients under the age of 5 with acute respiratory infections (ARIs) and performed a baseline questionnaire. The clinical and socioeconomic burden of RSV disease in primary care was evaluated by two follow‐up questionnaires completed by the parents of positive children on Days 14 and 30.

**Results:**

RSV laboratory‐confirmed cases were 37.8% of the total recruited ARI cases, with RSV subtype B accounting for the majority (65.4%) of RSV‐positive swabs. RSV‐positive children were younger than RSV‐negative ones (median 12.5 vs. 16.5 months). The mean duration of symptoms for all children infected by RSV was 11.47 ± 6.27 days. We did not observe substantial differences in clinical severity between the two RSV subtypes, but RSV‐A positive patients required more additional pediatric examinations than RSV‐B cases. The socioeconomic impact of RSV infection was considerable, causing 53% of children to be absent from school, 46% of parents to lose working days, and 25% of families to incur extra costs.

**Conclusions:**

Our findings describe a baseline of the RSV disease burden in primary care in Italy before the introduction of upcoming immunization strategies.

## 1 Background

Respiratory syncytial virus (RSV) is a ubiquitous pathogen that affects individuals of all age groups, with a significant impact on pediatric populations [1]. Understanding the epidemiology of RSV is essential in comprehending its burden on public health systems and devising effective preventive measures [2].

RSV exhibits a seasonal pattern of circulation, typically characterized by annual epidemics during the colder months in temperate regions. In these areas, RSV infections tend to peak during late autumn through early spring, with a notable increase in cases during the winter months [3].

RSV is further distinguished by its antigenic diversity, primarily classified into two major subtypes: RSV‐A and RSV‐B. Understanding the significance of these antigenic subtypes is crucial in assessing their role in the disease burden, transmission dynamics, and vaccine development [4]. Both RSV groups usually co‐circulate, even if RSV‐A is reported to be the predominant group during most seasons.

An alternating trend in terms of the prevalence of RSV‐A and RSV‐B in hospital‐admitted cases was reported in a study conducted in Italy, in the Lazio region, between 2015 and 2018 [5]. Most studies report no difference in clinical severity between children infected with RSV‐A and RSV‐B [6].

The onset of symptoms in an RSV infection typically occurs following an incubation period of 4–6 days. The influenza‐like symptoms caused by RSV may include shortness of breath, wheezing, cough, a sore throat, coryza, fever, and difficulties with feeding [2]. What is increasingly understood is that the impact of RSV extends beyond the acute phase. In particular, RSV infections that occur within the first year of life, even those not severe enough to warrant hospitalization, are linked to a higher likelihood of experiencing recurrent wheezing and an increased risk of developing asthma later on [1, 7, 8].

Among pediatric populations, infants and young children are particularly susceptible to severe RSV infections; in fact, this virus is a leading cause of bronchiolitis and pneumonia in children under the age of 5, resulting in substantial morbidity and mortality worldwide [9].

RSV is known as one of the most common causes of pediatric acute respiratory infections (ARIs), yet the global burden of the disease attributable to this virus is still unknown [10, 11, 12]. Although the scientific community is globally focusing on the surveillance of RSV transmission and the related hospital admission rate [13, 14], the specific burden of RSV in primary care settings remains inadequately characterized, especially in Italy, making it critical to investigate the associated clinical and socioeconomic impact [15].

The epidemiology of RSV exhibits geographical and temporal variability. This variability is influenced by factors such as climate, population density, healthcare infrastructure, and the presence of risk factors within specific communities [3]. Consequently, the burden of RSV may vary considerably between regions and even within different populations within the same country [16].

In temperate regions of the Northern Hemisphere, including Italy, virus diffusion generally occurs in the period spanning from October/November to March/April, with peak incidence in January/February, which partly overlaps with the influenza virus season [16, 17]. In Italy, restrictive measures for the prevention and control of the SARS‐COV‐2 virus pandemic—especially physical distancing, the use of face masks, and the discontinuation of in‐person teaching activities—are likely responsible for the reduced circulation of RSV and other respiratory pathogens during the 2020–2021 season detected by the Italian Influenza Surveillance Network (Influnet) system [18].

The current approaches available against RSV include a combination of general measures, such as hand washing and contact precautions, and the use of monoclonal antibodies in sensitive populations. Additionally, many vaccinations are being developed at different stages. Regarding passive immunization with monoclonal antibodies, for many years, palivizumab was the only monoclonal antibody authorized [19]. The EU recently approved the use of nirsevimab in both term and preterm infants [20, 21]. The RSV vaccine development pipeline includes several candidates, consisting of mRNA, live‐attenuated, subunit, and recombinant vector–based vaccines targeting different virus proteins. Abrysvo (Pfizer), a bivalent RSV prefusion F subunit vaccine, is the first vaccine approved for use on the market as a passive immunization agent for newborns by maternal administration during the third trimester of pregnancy [22].

The establishment of a universal pediatric RSV prevention strategy in Italy requires reliable precise epidemiological data on infection seasonality; these data will be critical for monitoring the impact of the immunization programs introduced.

The RSVComNet project [23], which started in the 2019–2020 season, has provided a platform aimed to assess the clinical and socioeconomic burden of RSV, particularly among children under 5 years of age in primary care settings, across multiple European countries [24].

As suggested by Azzari et al. [2], a strengthening of community‐based epidemiological surveillance of RSV infections in the Italian pediatric population was much needed.

Our study, originating from the Italian arm of the RSVComNet multicountry study, seeks to further explore the nature of the RSV disease burden in Italy's primary care setting and to describe the differences in RSV circulation patterns, risk factors, and clinical impact on the pediatric population within five Italian regions during the 2022–2023 winter season.

## 2 Methods

During the 2022–2023 winter season, 55 primary care pediatricians from five Italian regions (Lazio, Liguria, Lombardy, Apulia, and Tuscany) participated in the community‐based surveillance of the RSVComNet Italy study. In Italy, pediatric primary care up to 6 years of age is provided exclusively by primary care pediatricians [25].

In our study, pediatricians were recruited on a voluntary basis and given specific instructions to carry out nasal swab tests on all patients under the age of 5 who exhibited ARIs between Week 44 of 2022 and Week 13 of 2023. An ARI case was defined following the WHO‐ECDC case definition: “sudden onset of symptoms and at least one of the following four respiratory symptoms ‐cough, sore throat, shortness of breath, coryza‐ and a clinician's judgment that the illness is due to an infection” [26].

At the time of enrollment (referred to as T0), after obtaining informed consent from the participants, we collected detailed information about the clinical condition of each child and collected a nasopharyngeal swab. This swab was then analyzed to detect RSV genome by using multiplex RT‐qPCR (Allplex Respiratory Full Panel Assay, Seegene, South Korea), which involved a panel capable of detecting 27 different respiratory viruses and bacteria. We also collected data regarding the child's flu vaccination status at T0.

**FIGURE 1:**
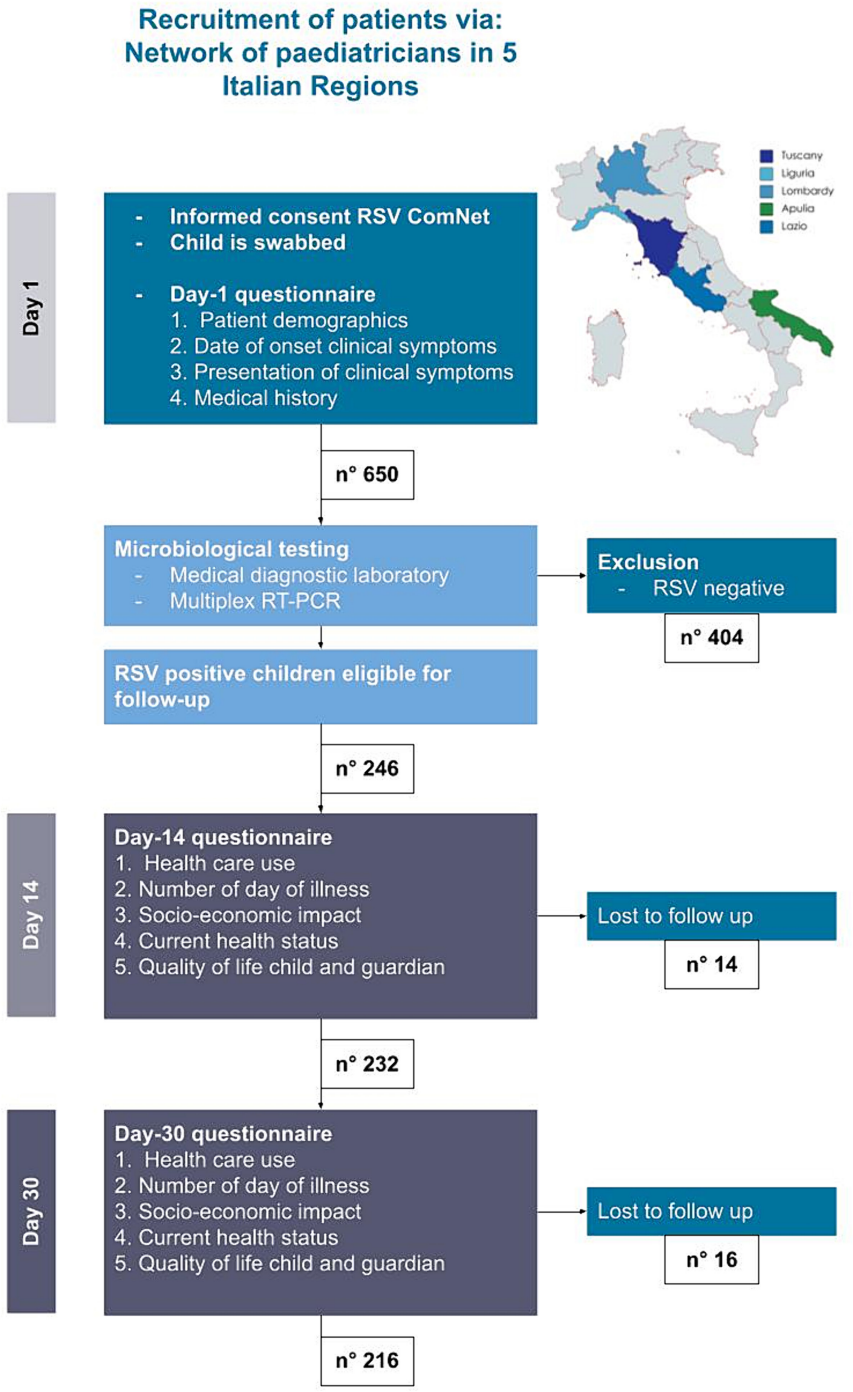
Flowchart: Disease burden protocol used in Italy RSV ComNet III. Children are selected via a network of pediatricians.

The overall study design is illustrated in Figure 1 in the form of a flowchart. Subsequently, the parents of children who tested positive for RSV were contacted via telephone and were requested to complete two follow‐up questionnaires on Day 14 and Day 30 after diagnosis. These questionnaires gathered information on the clinical and socioeconomic impact of the RSV infection (referred to as T14 and T30 questionnaires).

To represent RSV epidemiological trends in the five different regions, an epidemic curve was constructed using the R package EpiCurve. Socio‐demographic and clinical differences between children testing positive for RSV and the one testing negative were analyzed using Wilcoxon test for continuous variables and chi‐square test for categorical variables. Fisher's exact test was used instead of chi‐square test for categorical variables with small sample sizes (at least one category < 5). Furthermore, we conducted ANOVA and chi‐square tests to assess socio‐demographic and clinical differences among RSV cases in the five regions.

Only statistically significant results with a *p* value < 0.01 were considered for further investigations. All data analysis was conducted using R software.

## 3 Results

The 55 pediatricians enrolled have an estimated 18,500 assisted population of children under 5 years of age, which corresponds to 2% of the total population ≤5 years of age in the five regions. The total Italian population in this age group was around 2.2 million in 2022 [27], and 40% of those children lived in the five regions included in our study.

**FIGURE 2:**
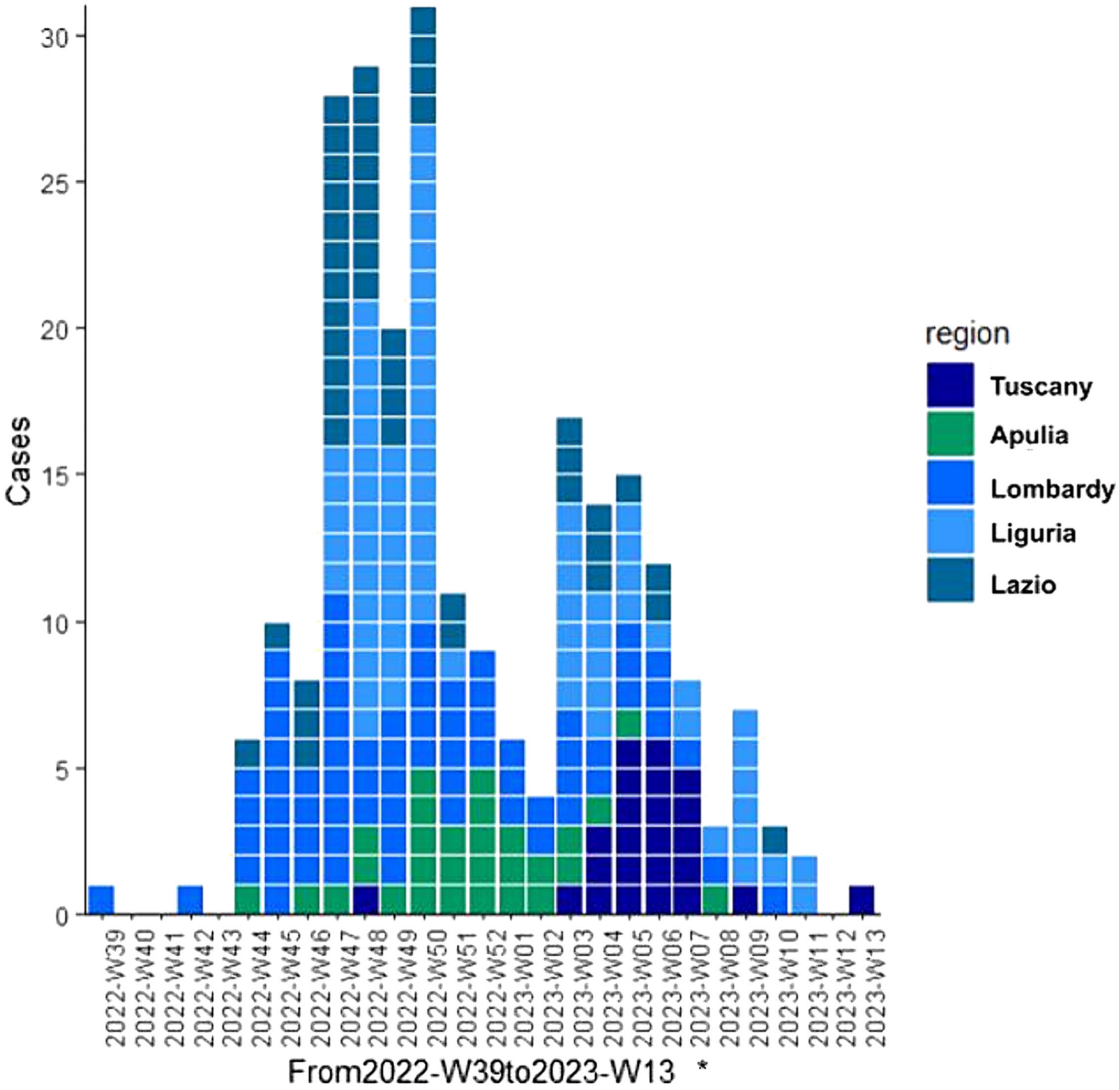
Epidemiological trend of RSV in the 2022–2023 winter season among the five participating Italian regions. *Tuscany started systematic enrollment of patients on W03‐2023. Lombardy started on W39‐2022. All the other regions started on W42‐2022.

The total number of children enrolled in the study was 650 (3.5% of the estimated assisted population under 5 years old), as they met the eligibility criteria for ARI. A total of 246 (37.8%) of the 650 children with ARI were RSV positive. The first peak in the epidemiological trend of RSV cases was reported in Week 47 of 2022, as shown in Figure 2, with cases observed in Lazio, Lombardy, and Liguria. The second and significantly lower peak in late January corresponds to the delayed start of recruitments in Tuscany. In fact, due to logistical reasons, only two children were recruited at the end of 2022 in Tuscany, although the systematic enrollment of children started in January 2023 (W03‐2023). The trend of the weekly proportion of RSV‐positive samples is reported in Data S1.

The demographic and clinical characteristics of the RSV‐positive and RSV‐negative children are described in Table 1.

**TABLE 1:** Demographics and baseline characteristics of RSV‐positive and RSV‐negative children. Variables with a * have a statistically significant difference between RSV‐positive and RSV‐negative groups.

Patient characteristics	Number of RSV‐positive children (*n* = 246)	Number of RSV‐negative children (*n* = 404)	Total (*n* = 650)
Age in months* (median, IQR) (mean, SD)	12.5 (6–22)	16.5 (8–30.25)	15 (8–28)
	16.43 ± 13.53	21.32 ± 16.35	19.47 ± 15.52
Age groups (*n*, %)			
0–5 months	52 (21.1%)	67 (16.6%)	119 (18.3%)
6–11 months	61 (24.8%)	71 (17.6%)	132 (20.3%)
0–11 months*	113 (46%)	138 (34.1%)	251 (38.6%)
12–23 months	74 (30%)	121 (30%)	195 (30%)
24–59 months*	59 (24%)	145 (35.9%)	204 (31.4%)
Birth weight gr. (median, IQR) (mean, SD)	3270 (3000–3555)	3210 (2900–3506)	3240 (2950–3548)
	3251 ± 547	3186 ± 547	3212 ± 548
Boys (*n*, %)	125 (50.8%)	202 (50%)	327 (50.3%)
Prematurity (*n*, %)	14 (5.7%)	38 (9.4%)	66 (10.1%)
Presence of chronic condition (*n*, %)			
Respiratory disease	9 (3.7%)	15 (3.7%)	26 (4%)
Congenital heart disease	4 (1.6%)	1 (0.2%)	5 (0.8%)
Immunocompromised	1 (0.4%)	1 (0.2%)	2 (0.3%)
Atopy	26 (10.6%)	37 (9.1%)	63 (9.7%)
Vaccination status (*n*, %)			
Influenza vaccination this season (*n*, %)	48 (19.5%)	97 (24%)	145 (22.3%)
RSV subtype			
RSV‐A	59 (24%)	0	59 (9.1%)
RSV‐B	161 (65.4%)	0	161 (24.7%)
RSV‐A + RSV‐B	2 (0.8%)	0	2 (0.3%)
Not available	24 (9.8%)	0	24 (3.7%)

RSV‐positive children are younger than RSV‐negative; this difference is statistically significant (Wilcoxon test, W = 41,125, *p* < 0.001).

The antigenic subtype of RSV that caused an infection was more frequently the RSV‐B (65.4%), with statistically significant differences among the five regions (chi‐square statistics: X^2^ = 35.24, *p* < 0.00001). The RSV‐B antigenic subtype was more prevalent than RSV‐A in every region in our study, except for Lombardy where RSV‐A and RSV‐B were almost equally represented, with 56.4% for subtype A and 43.6% for subtype B.

After performing chi‐square tests or Fisher's tests for categorical variables and ANOVA for age and birth weight, no statistically significant differences were observed in the basal characteristics of children infected by RSV among the five regions, except for atopy, which was most frequently observed among children in Apulia and Tuscany Fisher's test (*p* < 0.001). A comprehensive description of baseline patients' characteristics divided per region is provided in Table 2.

**TABLE 2:** Demographics and baseline characteristics of RSV+ children in five Italian regions. Variables with a * have a statistically significant difference among regions.

Patient characteristics	Number of subjects enrolled Lazio: 122	Number of subjects enrolled Liguria: 203	Number of subjects enrolled Lombardy: 164	Number of subjects enrolled Apulia: 65	Number of subjects enrolled Tuscany: 96
	Number of RSV+ Lazio: 70 (57.3%)	Number of RSV+ Liguria: 59 (29.1%)	Number of RSV+ Lombardy: 55 (33.5%)	Number of RSV+ Apulia: 38 (58%)	Number of RSV+ Tuscany: 24 (25%)
Age in months (median, IQR) (mean, SD)	12 (6.25–21.75)	13 (8–29)	14 (7.5–21)	12 (5–23.5)	9.5 (5.75–17.25)
	16.57 ± 13.64	18.74 ± 15.41	18.02 ± 11.92	16.24 ± 14.68	12 ± 8.95
Age groups (*n*, %)					
0–5 months	16 (23.2%)	9 (15.3%)	8 (15.1%)	13 (34.2%)	6 (25%)
6–11 months	17 (23.2%)	16 (27.1%)	14 (26.4%)	6 (15.8%)	8 (33.3%)
0–11 months	33 (46.3%)	25 (42.4%)	22 (41.5%)	19 (50%)	14 (58.3%)
12–23 months	21 (30.4%)	16 (27.1%)	22 (39.6%)	9 (23.7%)	6 (25%)
24–59 months	16 (23.2%)	18 (30.5%)	11 (18.9%)	10 (26.3%)	4 (16.7%)
Birth weight (median, IQR) (mean, SD)	3260 (3000–3447.5)	3230 (2900–3570)	3385 (3045–3562.5)	3262,5 (2925–3550)	3448 (3007.5–3600)
	3266 ± 437	3127 ± 890	3354 ± 628	3242 ± 571	3295 ± 603
Boys (*n*, %)	39 (55.7%)	27 (45.7%)	27 (49.1%)	18 (47.4%)	14 (58.3%)
Prematurity (*n*, %)	3 (4.3%)	7 (11.9%)	0	2 (5.3%)	2 (8.3%)
Presence of chronic condition (*n*, %)					
Respiratory disease	6 (8.6%)	2 (3.4%)	0	1 (2.6%)	0
Congenital heart disease	1 (1.4%)	3 (5%)	0	0	0
Immunocompromised	0	1 (1.7%)	0	0	0
Atopy*	6 (8.6%)	4 (6.8%)	0	11 (28.9%)	5 (20.8%)
Vaccination status (*n*, %)					
Influenza vaccination this season (*n*, %)	16 (22.8%)	17 (28.8%)	5 (9.1%)	8 (21.1%)	2 (8.3%)
RSV subtype					
RSV‐A*	9 (12.8%)	8 (13.5%)	31 (56.4%)	4 (10.5%)	7 (29.2%)
RSV‐B*	37 (52.9%)	49 (83.1%)	24 (43.6%)	34 (89.5%)	17 (70.8%)
RSV‐A + RSV‐B	0	2 (3.4%)	0	0	
Not available	24 (34.3%)	0	0	0	

Differences in the rate of flu vaccination were observed comparing RSV‐positive (19.5% vaccinated against flu) and RSV‐negative children (24% vaccinated against flu), despite not being statistically significant. Similarly, the number of RSV‐positive children that were vaccinated against flu varied comparing the five regions, but this result was not significant.

RSV‐positive children were followed up for 30 days with one questionnaire on Day 14 and one on Day 30. Few children were lost at follow‐up, specifically 14 (5.7%, 14/246) at T14 and 16 at T30 (6.9%, 16/216).

On Day 14, 115/232 children (49.6%) were still symptomatic, whereas on Day 30, 37/216 children (17.1%) had still persistent symptoms. The frequency of the various symptoms reported at different times is specified in Table 3.

**TABLE 3:** Clinical symptoms reported at baseline, T14, and T30.

Clinical symptoms (*n*, %)	Baseline *N* = 246	Day‐14 *N* = 232	Day‐30 *N* = 216
Shortness of breath	131 (53.2%)	5 (2.1%)	1 (0.4%)
Wheezing	110 (44.7%)	4 (1.7%)	2 (0.9%)
Wet cough	142 (57.7%)	39 (16.8%)	9 (4.2%)
Dry cough	126 (51.2%)	38 (16.4%)	18 (8.3%)
Sore throat	32 (13.0%)	5 (2.1%)	0
Coryza	200 (81.3%)	56 (24.1%)	18 (8.3%)
Fever ≥ 38°C	146 (59.3%)	7 (3.0%)	1 (0.5%)
Feeding difficulties	90 (36.6%)	14 (6.0%)	2 (0.9%)

The mean duration of symptoms for all children infected by RSV was 11.47 ± 6.27 days. The duration of symptoms for children positive for RSV‐A was 10.58 ± 5.45, whereas that for RSV‐B was 12.26 ± 6.83. The *t*‐test performed did not show the difference in illness duration to be statistically significant. The duration of symptoms was lower in the Lazio region (9.5 ± 3.6 days), as compared with the other areas (Liguria: 12.6 ± 7 days, Lombardy: 12.4 ± 7 days, Apulia: 12.5 ± 7.9 days, Tuscany; 11.6 ± 6.5 days). The ANOVA test yielded a significant result (*p* < 0.05).

In the overall period between the day of diagnosis and Day 30, 125/232 (53.8%) RSV‐positive children needed further pediatric medical examinations, with a mean number of 2.53 ± 2.84 examinations; 102 children (43.9%) needed extra phone/email pediatric consultations, with a mean number of 2.82 ± 2.76 consultations.

In the same period, 20/232 children (8.6%) needed to consult a different medical specialist, such as a pneumologist, a cardiologist, a dermatologist, a general practitioner, or a private pediatrician; 29/232 children (12.5%) had an emergency department access, with seven of them (3%) hospitalized, for an average length of stay of 4.7 ± 2.2 days. One of them (0.4%) was admitted to the ICU.

A total of 123/232 children (53%) had an absence from day‐care/school for an average duration of 13.02 ± 7.58 days.

Moreover, 46.1% (107/232) of parents had to ask for days off because of their children's illness for a mean duration of 7.92 ± 5.25 days.

Extra costs were reported by 59/232 (25.4%) parents, which included babysitting costs in 9/59 cases (15.3%), purchase of medicines in 52/59 (88.1%), or purchase of medical devices in 4/59 (6.8%).

In our study, we did not observe substantial differences in clinical severity between different RSV subtypes. However, it is worth noting a statistically significant result regarding the proportion of RSV‐A patients requiring additional pediatric examination at T30. Specifically, 29% of RSV‐A patients (11 out of 38) required further evaluation, whereas only 11% of RSV‐B patients (16 out of 142) needed such examination. The statistical analysis using chi‐square statistics yielded a value of X^2^ = 7.35, with a *p* value less than 0.01, indicating the significance of this difference (Table 4).

**TABLE 4:** Severity outcomes at T14 and T30 for RSV‐A and RSV‐B subgroups. Patients with missing subtype characterization and with positivity for both A and B subtypes were excluded. Variables with a * have a statistically significant difference.

	Follow‐up	RSV‐A T14 *n* = 53 T30 *n* = 38	RSV‐B T14 *n* = 157 T30 *n* = 142
Persistence of symptoms	T14	28	71
	T30	7	26
Further pediatric examination*	T14	36	109
	T30*	**11**	**16**
Emergency department access	T14	7	16
	T30	3	2
Hospitalization	T14	2	2
	T30	2	0
Complications	T30	4	9

*Note:* Bold indicates statistically significant variable.

RSV‐positive children received drug treatment in 89.7% of cases (208/232). The most used drugs were bronchodilator inhalers (116/232, 50%), followed by corticosteroid inhalers (69/232, 29.7%), paracetamol (61/232, 26.3%), and antibiotics (54/232, 23.3%). Antibiotic consumption was reported to be different in the five regions (X^2^ = 20.77, *p* < 0.01). Liguria was the region that reported the highest antibiotic prescription rate among RSV‐positive children (22/56, 39.3%), followed by Tuscany (9/23, 39.1%), Apulia (11/38, 28.9%), Lazio (8/67, 11.9%), and Lombardy (4/47, 8.5%). There were no significant differences in the utilization of bronchodilator and corticosteroid inhalers across the five regions. Conversely, the utilization of paracetamol displayed significant variability (X^2^ = 18.90, *p* < 0.001). Specifically, Tuscany had the lowest paracetamol usage, with only 4.3% (1/23) of cases receiving it. In contrast, Lombardy reported the highest paracetamol consumption, with 46.8% (22/47) of cases using this medication. Lazio followed closely with 29.9% (20/67) of cases using paracetamol, whereas Liguria reported 17.9% (10/56) usage. Apulia fell in between, with 21.1% (8/38) of cases receiving paracetamol.

Other therapies were also employed but were less often recommended, such as cortisone tablets (46/232, 19.8%), nonsteroidal anti‐inflammatory medications (20/232, 8.6%), nasal sprays (12/232, 5.2%), and cough syrups (2/232, 0.9%).

The most frequently reported complications among RSV‐positive patients were pneumonia with seven cases, otitis in four patients, and bronchiolitis in two children.

## 4 Discussion

The public health measures adopted against COVID‐19 pandemic significantly influenced the epidemiology of RSV [28, 29, 30]. This has been described in 2021, when Italy suffered a seasonal shift of RSV epidemic, with a beginning in August and a delayed peak in November [31].

Therefore, the mitigation of restrictive measures in Italy during the 2022–2023 winter season has been crucial to observe the characteristics of the RSV re‐emergence, its transmission, and seasonal trend. The epidemiological changes of RSV highlight the importance of seasonal surveillance and greater knowledge of the RSV burden in the community. This would allow the healthcare services to face the challenge of an appropriate preparation that meets the demands of RSV infections.

We started data collection in October (W42‐2022), and our study lasts 21 weeks in total. We observed a peak of RSV positivity starting in late November, between Weeks 47 and 50 of 2022, which encloses almost 50% of RSV‐positive cases of our sample. With little variation, the peak of cases occurs in this time frame in all regions (Lombardy and Lazio first, then Liguria, and lastly Apulia); due to logistical issues, the recruitment of patients was delayed in Tuscany, and as a result, the region's peak was postponed until Week 5 of 2023.

Nevertheless, our results show a change in the seasonal epidemic pattern of RSV, compared to the pre‐Covid era, an early peak in November. These data are in line with recent literature from Italy as well as from other European countries [32, 33].

The causes of the seasonal patterns and the changes in viral circulation throughout time are still unknown. Several theories have been postulated, contemplating the interaction and connection between multiple factors including seasonal variations in virus survival and transmissibility, physiology of the host, and the social behavior that fluctuates with the seasons and, in particular, after the epidemic [34].

Although seasonal and local variations in the preponderance of RSV subtypes A and B have been recorded, the literature provides evidence about the seasonal co‐circulation of both subtypes and the usual predominance of one subgroup in each season. The pattern of predominance is still evolving with seasonal and local variations, although it is clear that RSV‐A and RSV‐B alternately predominate, with a higher percentage of RSV‐B over RSV‐A infections over the past 10 years [4].

The differences in the prevalence of RSV antigenic subtypes among regions confirm a different epidemiology of RSV in different areas of Italy in the 2022–2023 season, which is not associated with baseline patient demographic and clinical characteristics. Our study found that subtype B was predominant in Italy during the 2022–2023 season, being observed in 65.4% of positive cases, whereas subtype A was observed in 24% of cases. Only two children had an RSV‐A and RSV‐B coinfection, although data on the subtype were not available for 24 children from the Lazio region. All the participating regions showed comparable proportions of both subtypes, with subtype B clearly dominating in all of them except for the Lombardy region, where there was roughly equal distribution of both subtypes. This is in line with other studies performed in the same season in other European countries, whereas the previous season showed the predominance of RSV‐A subtype [35].

Literature results are contradictory regarding the severity of the disease caused by the two subtypes of RSV. Due to the co‐circulation of multiple different strains throughout any given RSV season, some studies might not achieve the statistical power to identify significant differences in illness severity [29]. Our results showed a significant difference between subtypes in the clinical course of disease, with almost 30% of RSV‐A patients requiring further medical evaluation during the 30 days after the diagnosis, against about the 10% of RSV‐B patients who needed it. The literature reports that in the past seasons in Italy, RSV‐A viruses have been detected more frequently among patients with ARIs compared to RSV‐B viruses [36], regardless of age, prematurity, and other risk factors. Several studies suggested that RSV‐A infections are more clinically severe [37, 38].

In contrast, little research has shown the opposite [39], and some studies were utterly unable to find any relevant correlation between subtype and disease severity [40, 41]. These contradictions of findings emphasize the need for active surveillance of the RSV subtypes' circulation and the need to assess the pathogenic characteristics of the different genotypes causing moderate and severe RSV infections. In order to properly incorporate the more virulent strains into future vaccines, it may also be necessary to better understand the relationships between subtypes, genotypes, and disease severity. Monitoring the molecular evolution of RSV as well as its temporal and geographic differences may be crucial to help inform the development of targeted public health prevention strategies [42].

Comparing the RSV ComNet data with the ones provided by Influnet, the national influenza‐like illness (ILI) surveillance system [43], it appears that the RSV ComNet study detected a comparable proportion of RSV infections, both in children under 2 years of age (41.9% for RSVComNet and 49.1% for Influnet) and in children between 2 and 4 years of age (32.9% for RSV ComNet and 22.3% for Influnet). The difference in RSV detection rate could be explained by the different case definition used by these two surveillance systems (ARI vs. ILI).

Our study examined the presence of various socio‐demographic risk factors within the enrolled patients, in particular those that are quite consistently reported in literature such as age, prematurity, and preexisting health conditions (chronic lung disease, congenital heart disease and atopy), to explore the strength of an association with ARI diagnosis and RSV positivity.

Our data confirmed that younger children are those most at risk of infection by RSV. In fact, the age of RSV‐positive children was lower than that of negative children (median age 12.5 vs. 16.5 months) [44].

Unlike previous studies [9, 10, 11, 12], which focused mainly on the RSV‐associated morbidity, mortality, and hospitalization burden, our study represents a unique source of data for the evaluation of the RSV burden in primary care settings after the pandemic. The questionnaires' results showed that 71.4% of RSV‐positive children needed further medical consultation by the pediatrician, either in person or via phone/email, and 20.8% consulted another medical specialist and/or had access to the emergency department, describing a significant impact on the primary care system.

Interestingly, our results highlighted a common issue regarding inappropriate antibiotic prescription, with almost a quarter of RSV‐positive children who have received antibiotic treatment. Our data confirm what has already been published in other studies regarding the wide overprescription of antibiotics for respiratory disease in primary care [45], with about one‐third of all antibiotic prescriptions for ARIs in children being inappropriate [46].

The prescription of antibiotics was particularly high in the Tuscany and Liguria regions, with almost 40% RSV‐positive children receiving at least one antibiotic at T14, whereas in Lazio and Lombardy, approximately 10% of children reported antibiotic utilization. It is worth noting that the regions in which fewer antibiotics were consumed (Lombardy and Lazio) are the same that reported a higher utilization of paracetamol, 46.8% and 29.9%, respectively. This significant regional variation in antibiotic prescription likely reflects variations in local guidelines or interventions employed to contrast antimicrobial resistance and differences in pediatricians' training and experience influencing their prescribing behaviors. We do not have enough data to provide evidence‐based explanations of this finding, which merits further investigation to verify the appropriateness of prescription and to assess the antibiotic prescription policies existing in the various regions.

RSV‐positive children in the Lazio region were also the ones with the lowest duration of symptoms. The literature highlights geographical differences in the timing and duration of Influenza and RSV infections related to environmental risk factors, but these factors were not considered in our study and require further investigations.

It is widely recognized that RSV infections place a significant burden on affected families [47]. Through the questionnaires, our study also explored the socioeconomic impact of RSV infection in families, with nearly a half of parents who reported the need for days off at work, with an average duration of about 8 days, and with one in four parents reporting extra costs. It is therefore fundamental to raise the level of RSV awareness regarding the disease, symptoms, treatment, and prevention strategies among both healthcare professionals and parents.

Our study presents some limitations. One of these limitations is related to the community‐based surveillance method we used, which may have led to an underestimation of the number of severe RSV cases, as patients with more serious infections might bypass visiting a pediatrician and necessitate direct hospitalization. In addition, the enrollment of children might have been affected by a selection bias, because pediatricians may have more likely enrolled patients who they believed had a higher chance of being RSV cases. Our socioeconomic assessment captured the presence of additional economic burdens reported by families but did not delve into quantifying these costs; future studies should quantify RSV burden in primary care.

Our sample size was quite small, considering that the five regions participating in the study have a pediatric population aged less than 5 years that represents 40% of the Italian population in this age group. However, as previously reported, we only included the patients of 55 pediatricians willing to participate, thus limiting the number of possible ARI cases.

Moreover, the Tuscany regional center began data collection in late January, beyond the peak of the epidemic season. This may represent an issue in comparing trends between Tuscany and the other four regions.

## 5 Conclusions

Our study reveals significant insights into the RSV disease burden within primary care in Italy in the 2022–2023 season, highlighting a high prevalence of 37.8% RSV among ARIs in children under 5, with subtype B being the most common.

Interestingly, we found no significant differences in clinical severity between RSV subtypes, but RSV‐A‐positive cases required more pediatric examinations during the 30‐day follow‐up period. The implementation of RSV ComNet surveillance, employing the ARI definition across several European countries with a uniform methodology, bolsters the relevance of our findings within the broader European context of RSV research in primary care settings.

Understanding RSV epidemiology at different spatial scales can enhance the targeting and effectiveness of health interventions [47]. Despite the challenges in elucidating regional disparities, especially in Italy where a federated healthcare system is in place, the nuanced overview provided by our study emphasizes the importance of ongoing research and surveillance. This is particularly pertinent as emerging passive immunization methods and vaccines present new avenues for safeguarding young children [48, 49, 50].

## Author Contributions

**Michela Scarpaci:** Conceptualization; Investigation; Methodology; Project administration; Writing – original draft; Writing – review and editing. **Sara Bracaloni:** Conceptualization; Data curation; Project administration; Supervision; Validation; Writing – original draft; Writing – review and editing. **Enrica Esposito:** Conceptualization; Investigation; Writing – review and editing. **Luigi De Angelis:** Conceptualization; Formal analysis; Methodology; Writing – original draft. **Francesco Baglivo:** Conceptualization; Data curation; Methodology; Resources; Software; Visualization. **Beatrice Casini:** Conceptualization; Funding acquisition; Investigation; Writing – review and editing. **Donatella Panatto:** Project administration; Resources; Supervision; Writing – review and editing. **Matilde Ogliastro:** Investigation; Project administration; Resources. **Daniela Loconsole:** Conceptualization; Resources; Supervision; Validation; Writing – review and editing. **Maria Chironna:** Conceptualization; Project administration; Resources; Supervision; Writing – review and editing. **Elena Pariani:** Conceptualization; Investigation; Project administration; Resources; Writing – review and editing. **Laura Pellegrinelli:** Conceptualization; Project administration; Resources. **Elisabetta Pandolfi:** Funding acquisition; Project administration; Resources; Writing – review and editing. **Ileana Croci:** Formal analysis; Methodology; Software; Validation. **Caterina Rizzo:** Conceptualization; Funding acquisition; Investigation; Project administration; Resources; Supervision; Writing – review and editing. **RSVComNet Italy Working Group:** Funding acquisition; Project administration; Resources.

## Ethics Statement

The study was approved by the Ethical Committee “Comitato Etico di Area Vasta Nord Ovest (CEAVNO) per la Sperimentazione clinica” of the Tuscany Region, Italy (prot. 22871_Dini on 25/10/2022).

## Conflicts of Interest

LDA received travel reimbursement from Moderna. FB received an educational grant from AstraZeneca and travel reimbursement from Moderna. CR participated in Advisory Board and Expert scientific discussion for Seqirus, MSD, GlaxoSmithKline (GSK), Sanofi, and AstraZeneca. The other authors declare no conflicts of interest.

### Peer Review

The peer review history for this article is available at https://www.webofscience.com/api/gateway/wos/peer‐review/10.1111/irv.13282.

## Supporting information


**Data S1** Temporal trends in RSV positivity rates and children enrollment in our study.

## Data Availability

Data available on request due to privacy/ethical restrictions.
