# Supplementary figures and images for: RSV Disease Burden in Primary Care in Italy: A Multi‐Region Pediatric Study, Winter Season 2022–2023
Source: Influenza Other Respir Viruses. 2024 Apr 15;18(4):e13282. doi: 10.1111/irv.13282 (PMC11018906; doi:10.1111/irv.13282)

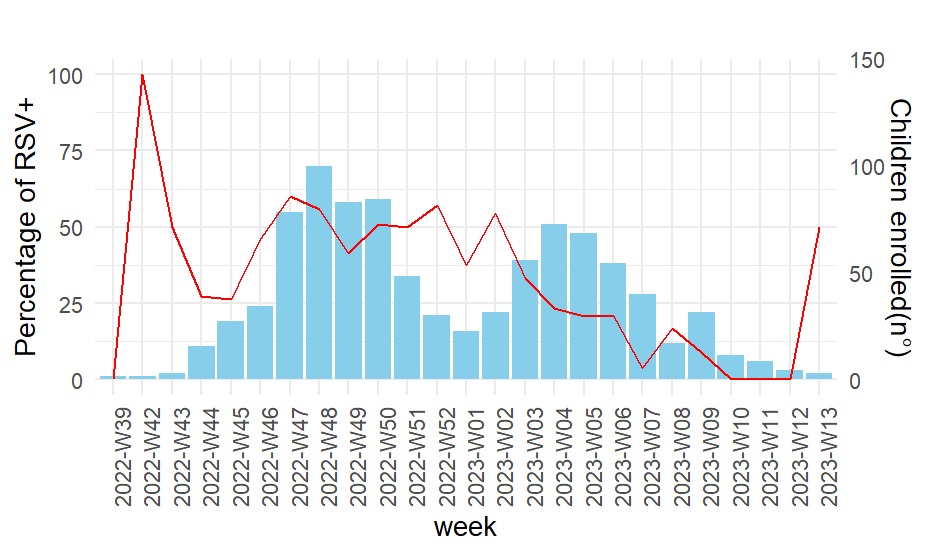

Supplement: Supplementary file 1 — Data S1 Temporal trends in RSV positivity rates and children enrollment in our study. [file IRV-18-e13282-s001.jpg]
